# Striatal Transcriptome Reveals Differences Between Cognitively Impaired and Unimpaired Aged Male Rats

**DOI:** 10.3389/fnagi.2020.611572

**Published:** 2021-01-08

**Authors:** Volker Korz, Christopher Kremslehner, Jovana Maliković, Ahmed Hussein, Daniel Daba Feyissa, Ionela-Mariana Nagelreiter, Roman Smidak, Roberto Plasenzotti, Florian Gruber, Gert Lubec

**Affiliations:** ^1^Proteomics Programme, Paracelsus Medical University, Salzburg, Austria; ^2^Department of Dermatology, Medical University of Vienna, Vienna, Austria; ^3^Center for Brain Research, Department of Molecular Neurosciences, Medical University of Vienna, Vienna, Austria; ^4^Institut für Labortierkunde, Medizinische Universität Wien, Vienna, Austria

**Keywords:** learning, memory, cognition, striatum, aging, GeneChip^TM^ microarray assay

## Abstract

Cognitive processes require striatal activity. The underlying molecular mechanisms are widely unknown. For this reason the striatal transcriptome of young (YM), aged cognitively impaired (OMB), and unimpaired (OMG) male rats was analyzed. The global comparison of transcripts reveal a higher number of differences between OMG and YM as compared to OMB and YM. Hierarchical clustering detects differences in up- and down-regulated gene clusters in OMG and OMB when compared to YM. In OMG we found more single genes to be specifically regulated in this group than in OMB when compared to young. These genes were considered as cognition specific, whereas genes shared in OMG and OMB were considered as age specific. OMB specific up-regulated genes are related to negative control of cell differentiation and transcription (Hopx), to phagocytosis (Cd202) and cell adhesion (Pcdhb21), whereas down-regulated genes are related to associative learning, behavioral fear response and synaptic transmission (Gabra5). OMG specific up-regulated genes are in the context of maintenance of transcription and estrogen receptor signaling (Padi2, Anxa3), signal transduction [Rassf4, Dock8)], sterol regulation (Srebf1), and complement activity (C4a, C4b). Down-regulated genes are related to lipid oxidation reduction processes (Far2) and positive regulation of axon extension (Islr2). These relations were supported by pathway analysis, which reveals cholesterol metabolism processes in both aged group and cholesterol biosynthesis specifically in OMG; adipogenesis and focal adhesion in OMB. In OMG glucuronidation, estrogen metabolism, inflammatory responses and TGF beta signaling where detected as specific for this group. Signal transduction of the sphingosine-1-phospate-receptor (S1P) receptor was the main pathway difference in the comparison of OMB and OMG with downregulated genes in the first group. This difference could also be observed in the OMB vs. YM comparison but not in the OMG vs. YM analysis. Thus, an up-regulation of cognition related genes could be observed in OMG compared to OMB rats. The S1P pathway discriminated between OMB and OMG as well as between OMB and OMG. Since this pathway has been described as essential for cognitive processes in the striatum of mice, it may, among steroid hormone signaling, significantly contribute to the maintenance of cognitive processes in OMG.

## Introduction

Striatal involvement in spatial learning is well-documented (Luine and Hearns, 1990; Lavoie and Mizumori, 1994; McDonald and White, 1994; Packard and Teather, 1997; Gengler et al., 2005). Torromino et al. (2019) found the cross-regional communication between hippocampus and the ventral striatum to play a critical role in spatial memory formation in mice after water maze training. Simultaneous activation of the dorsal striatum and the hippocampus is required for correct navigation in a double H maze in a cued environment (Gasser et al., 2020). Despite this long standing knowledge the underlying molecular mechanisms are largely unknown. Most transcriptomic and proteomic analyses were performed in the context of disease, drug addiction or pharmacology. Xu et al. (2007) found the striatum less responsive than other brain regions in terms of transcriptome changes related to age in mice. Genes were mostly regulated in the context of transcription regulation, protein synthesis and degradation and signal transduction. Similar gene expression profiles in the striatum and hippocampus have been found in NOS-1 (neural isofrom of nitric oxide synthase) knockdown animals which show impairments of spatial learning and memory including the holeboard test. Highly upregulated genes were related to gamma-Aminobutyric acid (GABA) -ergic signaling and the glucocorticoid receptor (GR). Martín-García et al. (2015) found genes related to extracellular signal-regulated kinase 5 (ERK5) signaling, cytokine signaling and protein ubiquitination in the ventral striatum of mice during extinction learning of operant conditioning.

Hamezah et al. (2018) profiled the proteome of the striatum in male rats at different ages, detecting only 5 proteins differently expressed with age. The affected pathway was the glutathione metabolism with increased expression of the proteins Glutathione S-transferase A3 (GSTA3) and Superoxide Dismutase 1 (SOD1) in 23 months old rats. However, rats were not tested in a cognitive task. Auditory learning in a shuttle box induced regulations of some synaptic proteins like Titin and Casitas B lineage lymphoma (CBL) in mice, the latter involved in protein degrading processes (Kähne et al., 2012). The same training induced also protein rearrangement in the striatum involving GABA and dopamine receptor signaling (Kähne et al., 2016).

Because of the small body of literature regarding age and cognition related effects upon gene expression in the striatum, we combined both aspects in the present study. Therefore, we compared young males with aged ones and divided the latter into bad (OMB) and good (OMG) performers, similar as in the study of De Risi et al. (2020) in mice. This approach provides information about age effects and should reveal mechanisms of cognitive maintenance during aging. The study was designed as a screening test involving a large number of genes. This should provide a basis for own follow up studies on a reduced number of here identified critical genes and pathways involving real time PCR and proteomic studies. The here presented results may also be of interest for other researchers in the field.

## Methods

### Animals

Animals were bred and maintained in the Core Unit of Biomedical Research, Division of Laboratory Animal Science and Genetics, Medical University of Vienna. One week before and throughout the experiment the animals lived in a separate experimental room and were housed individually in standard Makrolon cages filled with autoclaved woodchips at a temperature of 22 ± 2°C with a humidity of 55 ± 5% and 12 h artificial light/12 h dark cycle (light on at 7:00 a.m.). Individual housing should avoid social rank specific impacts on behavioral performance in the test. Tap water and food (R/M-H Ered II) diets from ssniff®, Soest, Germany) was provided at libitum. The study was carried out according to the guidelines of the Ethics Committee, Medical University of Vienna, and was approved by the Federal Ministry of Education, Science and Culture, Austria.

Three groups of male Sprague–Dawley male rats (8 animals per group) were tested for their transcriptome profile in the striatum: young males (YM), old males with bad (OMB) and good (OMG) memory. YM were 3 months old with an average weight of 440 g, aged rats were 20 months old with an average weight of 650 g. The animal samples represent a subgroup of animals from a previous study. In this study the effects of different diets on memory were studied. The here used animals were fed with the low energy diet as described in Maliković et al. (2018). In order to distinct between old male rats with impaired and unimpaired memory, a hole-board memory test was performed according to a previously described protocol (Maliković et al., 2018; Daba Feyissa et al., 2019). The spatial memory performance was evaluated based on Reference Memory Index (RMI) calculated as: (first + revisits of baited holes) / total visits of all holes). In order to compare individuals with similar levels of motivation, animals with <40 hole-visits in total over the 10 trials were excluded from the analysis. The individuals were considered as good performers (OMG) when RMI >the mean RMI value + standard deviation, and as bad performers (OMB) when RMI <the mean RMI value – standard deviation, with the mean value obtained from all animals included in the analysis over the evaluated training/testing period. To determine statistical significance of the differences between the two groups of old rats, the data was analyzed Graphpad-Prism 5 using a two way repeated measures ANOVA with age as one and training trial as the second factor, followed by a *post-hoc* comparison (Bonferroni) between groups. The significance was set at *p* < 0.05.

Four to six weeks after the behavioral test the animals were decapitated, the brains were rapidly removed and dissected on a Para Cooler (RWW Medizintechnik, Hallerndorf, Germany) at 4°C to obtain the striatum. The tissue was immediately stored at −80°C until transcriptomic analysis.

### RNA Purification

Total RNA was extracted from rat striatum tissue according to the “Purification of total RNA from animal and human tissue” protocol of the RNeasy Micro Kit (QIAGEN, Hilden, Germany).

In brief, the sample was frozen and shipped on dry ice. After adding 800 μl buffer RLT containing 1% beta-mercaptoethanol the tissue was disrupted and homogenized with Precellys CK14 ceramic beads (1 cycle of 20 s at 6,000 rpm) using a Precellys 24 Homogenisator (Bertin Corp., Rockville, MD, USA). Next the sample was centrifuged for 3 min at full speed and 350 μl of the cleared supernatant was transferred to a new tube. One volume of 70% ethanol was added and the sample was applied to a RNeasy MinElute spin column followed by an on-column DNase digestion and several wash steps. Finally total RNA was eluted in 14 μl of nuclease free water. Purity and integrity of the RNA was assessed on the Agilent 2100 Bioanalyzer with the RNA 6000 Nano LabChip reagent set (Agilent, Palo Alto, CA, USA).

### GeneChip^TM^ Microarray Assay

Sample preparation for microarray hybridization was carried out as described in the Applied Biosystems^TM^ GeneChip^TM^ Whole Transcript (WT) PLUS Reagent Kit User Guide (Thermo Fisher Scientific, Waltham, MA, USA). In brief, 200 ng of total RNA was used to generate double-stranded cDNA. 12 μg of subsequently synthesized cRNA were purified and reverse transcribed into single-stranded (ss) cDNA, whereas unnatural dUTP residues were incorporated. Purified ss cDNA was fragmented using a combination of uracil DNA glycosylase (UDG) and apurinic/apyrimidinic endonuclease 1 (APE 1) followed by a terminal labeling with biotin. 3,8 μg of fragmented and labeled ss cDNA were hybridized to Applied Biosystems^TM^ GeneChip^TM^ Clariom S rat arrays for 16 h at 45°C and 60 rpm in an Applied Biosystems^TM^ GeneChip^TM^ hybridization oven 640. Hybridized arrays were washed and stained in an Applied Biosystems^TM^ GeneChip^TM^ Fluidics Station FS450, and the fluorescent signals were measured with an Applied Biosystems^TM^ GeneChip^TM^ GeneChip Scanner 3000 7G System. Fluidics and scan functions were controlled by the Applied Biosystems^TM^ GeneChip^TM^ Command Console v4.3.3 software.

RNA extraction and sample processing were performed at a Genomics Core Facility, “KFB - Center of Excellence for Fluorescent Bioanalytics” (Regensburg, Germany; www.kfb-regensburg.de).

### Microarray Data Analysis

Summarized probe set signals in log2 scale were calculated by using the GCCN-SST-RMA algorithm with the Applied Biosystems^TM^ GeneChip^TM^ Expression Console v1.4 Software. After exporting into Microsoft Excel average signal values, comparison fold changes, significance *P*-values, hierarchical clustering, Venn-diagram and pathway-analysis were done with the transcriptome analysis console (V. 4.0.2.15, applied biosystems, Thermo Fisher Scientific). Probe sets with a student's *t*-test *P*-value lower than 0.05 were considered as significantly regulated.

## Results

### Behavior

**Figure 1:**
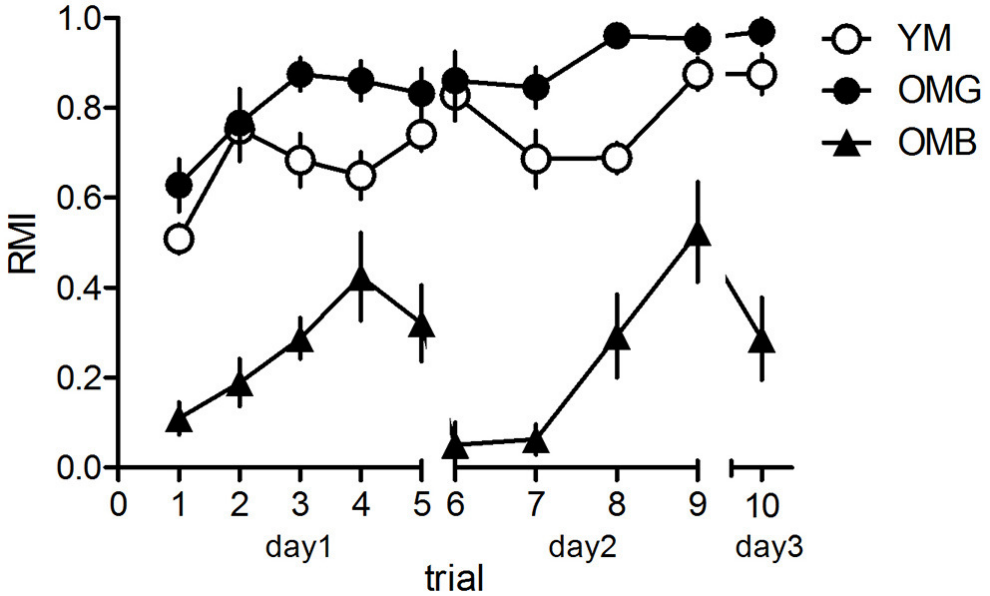
Behavioral results for the hole-board test. Young (YM) and old, cognitively unimpaired males (OMG) showed no differences in reference memory indices (RMI) over trials with the exeption of trial 8 where OMG performed better than YM. Both, YM and OMG exhibited higher RMI compared to cognitively impaired aged males (OMB) over trials with the exception of trial 4 where YM and OMB showed no difference. For statistics see the Results section. Given are the mean values and standard errors of the mean, *n* = 8 for each group).

The behavioral results are shown in Figure 1. The two-way ANOVA revealed a significant effect between groups (*F*_2,21_ = 54.42, *p* < 0.0001), a significant trial effect (*F*_9,189_ = 11.13, *p* < 0.0001) and a significant trial x group interaction (*F*_18,189_ = 6.34, *p* = 0.0005). *Post-hoc* tests revealed no significant difference between trial performance as compared between YM and OMG (*p* > 0.05, each) except for trial 8 with better performance in OMG (*t* = 2.90, *p* < 0.05) whereas OMB had significantly lower RMI compared to YM (*p* < 0.05) except for trial 4 with no difference (*t* = 1.29, *p* > 0.05). OMG rats show significantly higher RMI in all trials when compared to OMB (*p* < 0.05, each).

### Transcriptome

**Figure 2:**
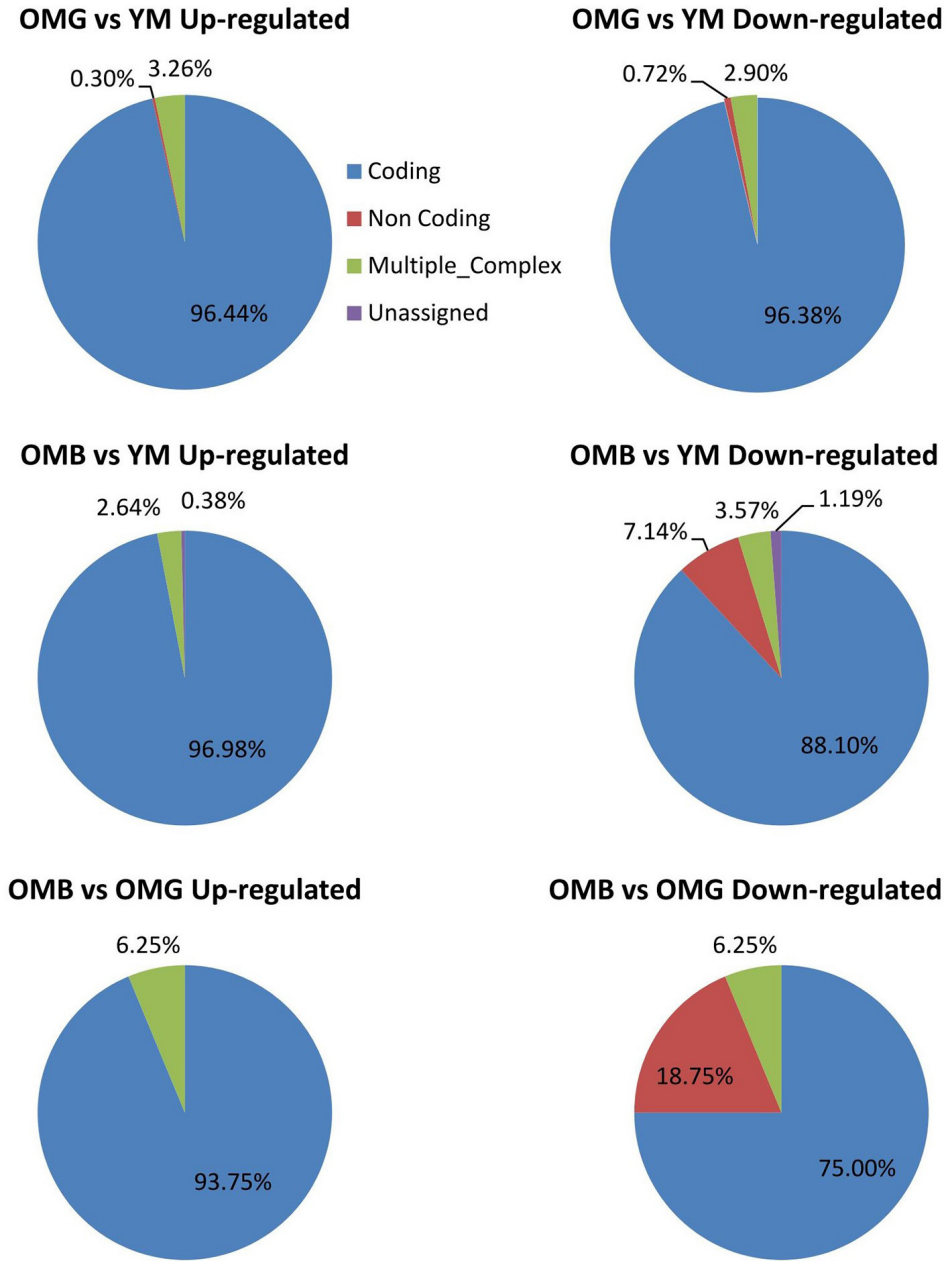
Summary of the transcriptome analysis. Characterization of regulated genes in the OMH vs. YM (upper panel), the OMB vs. YM (middle panel) and the OMB vs. OMG (lower panel) comparisons.

We found 367 significant differences in transcript amounts between OMB and YM and 610 between OMG and YM, whereas between OMG and OMB we found only 59 with a fold change of ≤-1.5 or ≥1.5 and a *p*-value ≤0.05. In the OMG vs. YM comparison most of the genes that differed between groups were coding (Figure 2). This was also true for the OMB vs. YM in upregulated genes whereas downregulated transcripts show a higher proportion of non-coding and unassigned genes. In the OMB vs. OMG comaparison the percentage of non-coding downregulated genes was even higher with 18.75%.

**Figure 3:**
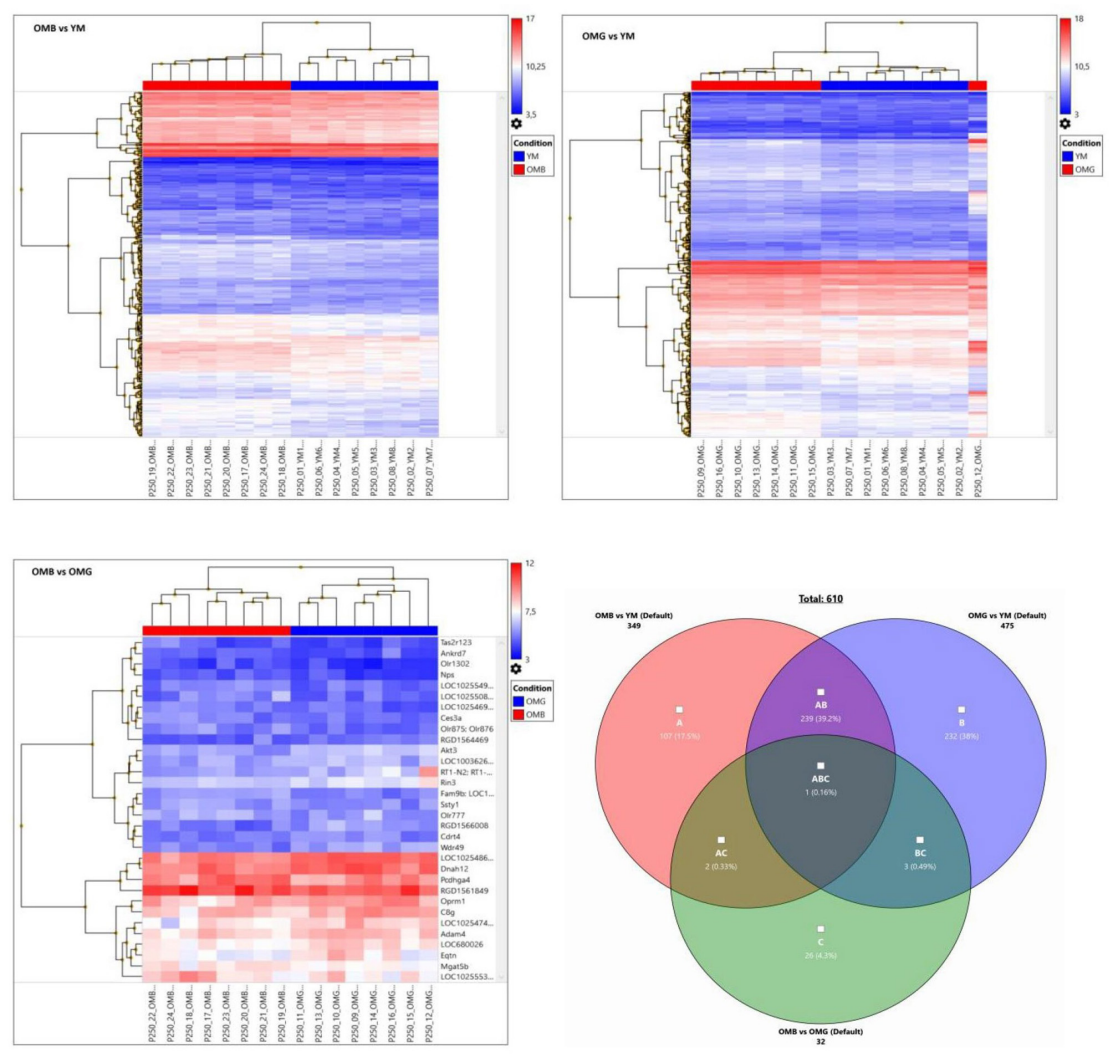
Hierarchical clusters of OMB vs. YM (upper left), OMG vs. (YM upper right) and OMB vs. OMG (lower left) comparisons for genes with a fold change of ≤-1.5 and ≥1.5 and *p* ≤ 0.05. Autoscale gives log2 signals beyond the middle in red and below in blue. Venn- diagram lower right, showing relations between groups.

The hierarchical clusters (Figure 3) revealed differences in certain gene clusters. In the upper part of Figure 2 OMG together with YM showed down-regulated clusters (genes Mgat4e, protein N-linked glycosylation, and Olr1555, sensory perception of smell:); (RGD1561318, histone lysin methylation, and Sult1c3, sulfur compound metabolic process); (Stox1, regulation of gene expression and, cellular response to nitrosative stress and Itgb3bp, integrin subunit beta 3 binding protein). Most remarkably one OMG animal did not cluster with OMG and not with YM.

**Figure 4:**
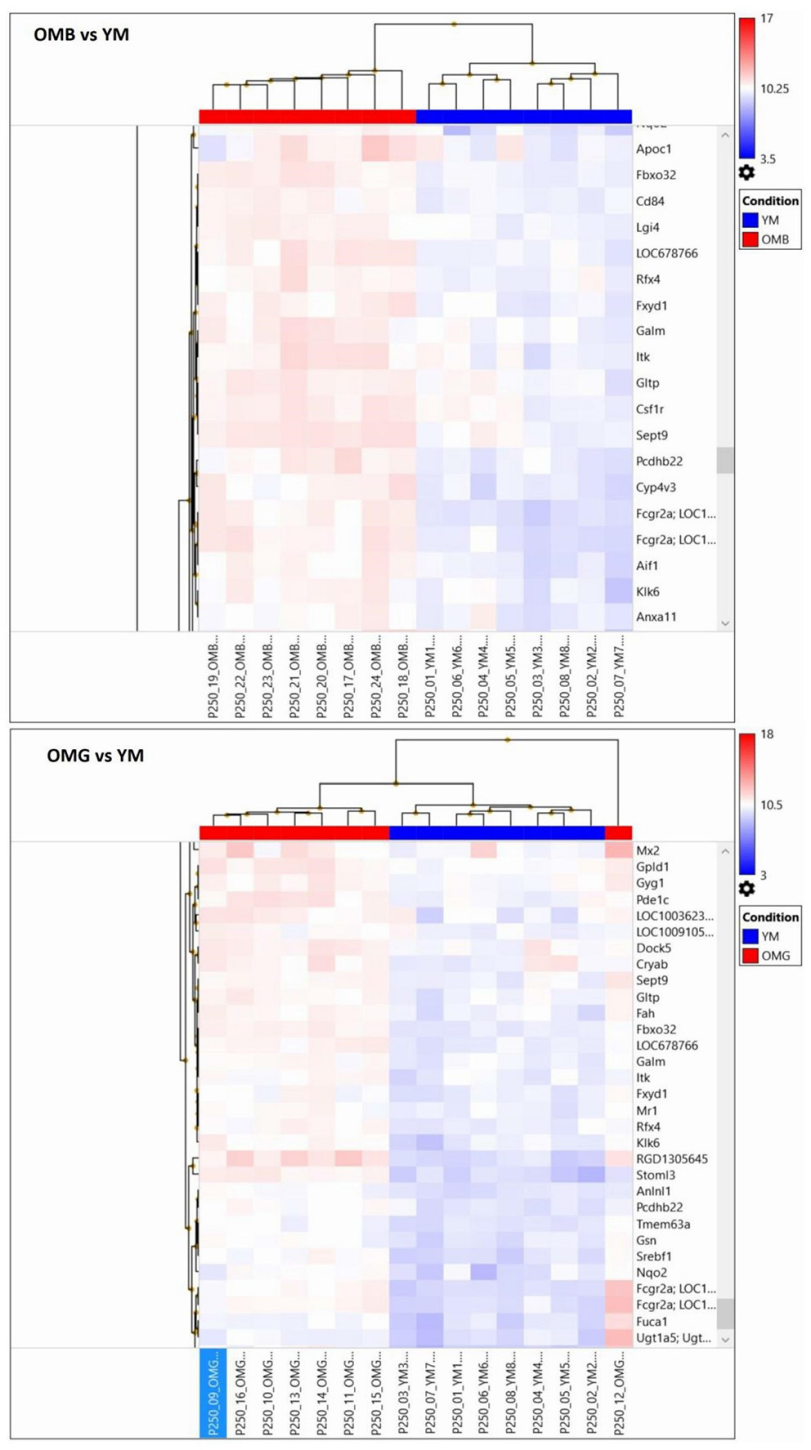
Random extraction of clusters with opposing log2 signals between compared groups in order to demonstrate within group variabilities for the OMB vs. YM (upper panel) and the OMG vs. YM (lower panel) comparisons.

For demonstration of individual heterogeneity a random subset of differently expressed genes between aged and young males were extracted and given in Figure 4. In OMB the variation between individual rats is lower than in OMG. In the OMG vs. YM analysis differences were found in clusters (Pde1c, phosphodiesterase 1c involved in cellular signaling, and LOC100362345, hypothetical protein LOC100362345), (RGD1305645, neuropeptide signaling pathway, positive regulation of corticosterone secretion, regulation of cell proliferation, and Stoml3, signal transduction), (Srebf1, sterol regulatory element binding transcription factor 1, and Nqo2, memory, oxidation-reduction process) with an up-regulation in OMG as compared to YM.

In the OMB vs. YM clusters (Itih3, inter-alpha trypsin inhibitor, and Cml3, histone acetylation); Zcchc24, zinc ion binding, and Tmem192, protein homodimerization activity), (S100b, calcium binding protein B, and Spock1, sparc/osteonectin, cwcv and kazal like domains proteoglycan 1) are upregulated. Specific cluster (Apoc1, cholesterol metabolic process, negative regulation of lipid metabolic process, and Fbxo32, F-box protein 32), (Cd84, Cd84 molecule and Lgi 4, leucine-rich repeat LGI family, member 4), (LOC678766, actin cytosceleton organization, and Rfx4, regulatory factor X4) are upregulated in OMB compared to YM.

In order to reduce the amount of genes and to figure out the most meaningful differences, the criterion was set to a fold cutoff at ≤-2 and ≥2 and *p* ≤ 0.05. The genes regulated in both comparisons of aged and young males are given in Table 1. Genes that are specifically regulated within one group are given in bold. These genes are considered to be cognition related, whereas shared genes are considered to be age related. In OMB vs. YM the Hopx, homeodomain-only; Slc9a, solute carrier family 9 member A9; Rt1-Ce13, RT1 class I, locus CE13 are upregulated in the first with the highest fold changes. In Table 2 the description and biological processes in which the OMB specific genes are involved are given. For most of the up-regulated genes biological processes are unknown, however the Hopx gene with the highest fold change is related to negative regulation of cell differentiation and transcription. Down-regulated genes are involved in cell adhesion and migration (Itgb6, Integrin alpha-6), associative learning and synaptic transmission gamma-aminobutyric acid type A receptor subunit alpha5 (Gabra5) and G protein coupled signal transduction.

**Table 1:** Comparison of old vs. young males, cognition-related genes (not shared between OMG and OMB) are given in bold.

**Up-regulated**			**Down-regulated**	
**OMB**	**OMG**	**OMG**	**OMB**	**OMG**
Ifi27	**F3**	Fcgr2a; LOC103693683; LOC498276; LOC100911825	Nr4a3	**Slc17a7**
Rnaset2	Lcp1		Sema3a	Nr4a3
**Hopx**	Ifi27		Neurod6	**Tbr1**
A2m	**Ctsz**	Cp	**Ccdc77**	Arc
**Slc9a3**	**Lgals3bp**	Pbld1	Nr4a1	Sema3a
Serpinb9	**Fbxo32**	**C4a; LOC100911579;** **LOC100360835;** **LOC102549354**	Arc	Nr4a1
Clec5a	A2m; LOC100911545		Per2	**Snx10**
**RT1-CE13**	**Rasgrp3**		**Itgb6**	Neurod6
Fcgr2a	Qdpr	RT1-Da	**Gabra5**	**Islr2**
Cp	**Rassf4**	Serping1	Egr2	**Far2**
**Aadat**	Serpinb9	Stoml3	Junb	Per2
**Cd302**	Rnaset2	Cdhr3	**Gpr63**	Egr2
Npc2	**Srebf1**	**Fcrl2**		**Ca10; Car10**
Bmp6	**Csf1**	Gfap		Junb
**Cryab**	**Padi2**	Fcgr2b		
Lcp1	Tmem176b	RGD1305645		
Mgst1	**Pld4**	Cd74		
**Pcdhb21**	LOC498276; LOC103693683; LOC100362543	Pla2g7		
Tmem176b	Fcgr2a	C3		
Stoml3	Bmp6			
Qdpr	**Fgl2**			
LOC498276; LOC103693683; LOC100362543	**Fam111a**			
LOC100910415	Irgm			
Irgm	**Smad9**			
Fcgr2a; LOC100912061	Mgst1			
**Agt**	Fcer1g			
Fcgr2a; LOC103693683; LOC498276; LOC100911825	**Cd180**			
Fcer1g	**LOC103689965; C4b; LOC100909666**			
Cd4	**A2m**			
Laptm5	**Dnah12**			
Pbld1	**Dock8**			
**Pcdhga4**	Npc2			
Serping1	Fcgr2a; LOC100912061			
Gfap	Clec5a			
Cdhr3	**Gbp2**			
RT1-Da	**RGD1309362**			
RGD1305645	Cd4			
Fcgr2b	**Anxa3**			
Pla2g7	LOC100910415			
C3	Laptm5			
Cd74	**Cyb5r2**			

*Fold change ≤-2 and ≥2, p ≤ 0.05. Genes are given in each group descending (from top to bottom) in fold change for up-regulated and ascending in fold change for down-regulated genes. Group specific genes are given in bold*.

**Table 2:** OMB specific regulated genes compared to YM, description and biological process (NCBI, gene catalog).

	**Description and biological function**
**OMB UP-REGULATED**	
Hopx	HOP homeobox, coding; heart development, negative regulation of cell differentiation and transcription by RNA polymerase II
Slc9a3	solute carrier family 9, subfamily A (NHE3, cation proton antiporter 3), member 3, coding
RT1-CE13	RT1 class I, locus CE13,coding
Aadat	aminoadipate aminotransferase, coding
Cd302	CD302 molecule, coding, phagocytosis
Cryab	crystallin, alpha B, coding
Pcdhb21	protocadherin beta 21, coding; cell adhesion
Agt	angiotensinogen (serpin peptidase inhibitor, clade A, member 8),coding
Pcdhga4	protocadherin gamma subfamily A, 4, coding; cell adhesion, multiple complex
**OMB DOWN-REGULATED**	
Ccdc77	coiled-coil domain containing 77, coding
Itgb6	integrin, beta 6, coding; cell adhesion, cell migration
Gabra5	gamma-aminobutyric acid (GABA) A receptor, alpha 5, coding; associative learning, behavioral fear response, chemical synaptic transmission
Gpr63	G protein-coupled receptor 63, coding; signaling pathway

The amount of specifically related genes in OMG rats is higher as compared to OMB (Table 3). Genes related to signal transduction (Rassf4), steroid membrane lipids regulation (Srebf1, Dock8, dedicator of cytokinesis 8; Cyb5r2, cytochrome B5 reductase 2), steroid (estrogenic) signaling and transcription maintenance (Padi2, Peptidyl Arginine Deiminase 2; Anxa3, Annexin A3), glucocorticoid response (Anxa3), and the activation and maintenance of the complement system (C4a, complement C4-A; C4b, complement C4-B) are upregulated. Down-regulated are gene transcripts involved in positive regulation of axon extension (Islr2, **i**mmunoglobulin superfamily containing leucine rich repeat 2) and lipid oxidation reduction process (Far2, fatty acyl-CoA reductase 2).

**Table 3:** OMG specific regulated genes compared to YM, description and biological process (NCBI, gene catalog).

	**Description and biological process**
**OMG UP-REGULATED**	
F3	coagulation factor III (thromboplastin, tissue factor), coding
Lgals3bp	lectin, galactoside-binding, soluble, 3 binding protein, coding
Fbxo32	F-box protein 32, coding
Rasgrp3	RAS guanyl releasing protein 3 (calcium and DAG-regulated), coding
Rassf4	Ras association (RalGDS/AF-6) domain family member 4, coding; signal transduction
Srebf1	sterol regulatory element binding transcription factor 1, coding
Csf1	colony stimulating factor 1 (macrophage), coding
Padi2	peptidyl arginine deiminase, type II, coding; cellular response to leukemia, inhibitory factor, chromatin mediated maintenance of transcription, intracellular estrogen receptor signaling pathway, histone citrullination
Pld4	phospholipase D family, member 4, coding; hematopoietic progenitor cell differentiation, phagocytosis
Fgl2	fibrinogen-like 2, coding
Fam111a	family with sequence similarity 111, member A, coding
Smad9	SMAD family member 9, coding
Cd180	CD180 molecule, coding
LOC103689965; C4b; LOC100909666	complement C4-like; complement component 4B, coding; inflammatory response (inferred), negative regulation of endopeptidase activity, activation of complement system
A2m	alpha-2-macroglobulin, coding;
Dnah12	dynein, axonemal, heavy chain 12; coding; ATP binding (inferred); ATPase activity (inferred), microtubule motor activity (inferred); ATP catabolic process (inferred)
Dock8	dedicator of cytokinesis 8, coding; cellular response to chemokine, dendritic cell migration, memory T cell profileration, negative regulation of T cell apoptotic processes, positive regulation of GPTase activity, small GPTase mediated signal transduction
Gbp2	guanylate binding protein 2, interferon-inducible, coding
RGD1309362	similar to interferon-inducible GTPase, coding; cellular response to interferon-beta, defense response
Anxa3	annexin A3, coding; defense response to bacterium, hippocampus development, phagocytosis, positive regulation of DNA-binding transcription activity, response to glucocorticoid, response to growth factor
Cyb5r2	cytochrome b5 reductase 2. Coding, oxidation reduction process, sterol biosynthetic process
C4a; LOC100911579; LOC100360835; LOC102549354	complement component 4A (Rodgers blood group); complement C4-like, coding
**OMG DOWN-REGULATED**	
Slc17a7	solute carrier family 17 (vesicular glutamate transporter), member 7, coding
Tbr1	T-box, brain, 1, coding
Snx10	sorting nexin 10, coding
Islr2	immunoglobulin superfamily containing leucine-rich repeat 2, coding; positive regulation of axon extension
Far2	fatty acyl CoA reductase 2, coding; lipid metabolic process, oxidation reduction process
Ca10; Car10	Protein Ca10; carbonic anhydrase 10, coding

The next step was the identification of pathway differences between groups (Table 4). Here again the criterion was set at ≤-1.5 and ≥1.5 and *p* ≤ 0.05 in order to detect also weaker differences in order not to miss pathways because of false negative results. Between OMB and OMG rats we found only one pathway to be different, that is the signal transduction of S1P receptor. This difference could also be observed between OMB and YM, but not between OMG and YM. Complement activation and activity appeared in bot aged groups compared to YM. Adiposegenesis, focal adhesion, B cell receptor signaling and Eicosanoid synthesis were specific for OMB, whereas glucoronidation, estrogen metabolism, cholesterol synthesis, Transforming growth factor beta (TGF beta) signaling, monoamine G protein-coupled receptors (GPCRs) and metapathway biotransformation were specific for OMG when compared to YM.

**Table 4:** Pathway analysis (www.wikipathways.org) for aged vs. young and between aged groups.

**OMB vs. YM**	**Total**	**Up**	**Up**	**Down**
Prostaglandin synthesis and regulation	6	6	Anxa3,Anxa5,Ptgs1,Ptgds,Anxa1,Anxa4	
Statin pathway	4	3	Lcat,Abca1,Apoc1	Lpl
Cholesterol metabolism	4	2	Srebf1,Apoc1	Hmgcs1,Lpl
Serotonin and anxiety	3	1	Plek	Htr2a,Arc
Hypertrophy model	3	1	Cyr61	Nr4a3,Dusp14l1
**Adipogenesis**	7	3	Srebf1,Agt,Spock1	Wnt10b,Lpl,Klf5,Egr2
Spinal cord injury	6	4	Tnfsf13,Fcgr2a,Anxa1,Gfap	Nr4a1,Egr1
Oxidative stress	3	2	Nqo1,Mgst1	Junb
Complement and coagulation cascades	4	4	F3,C3,A2m,C1s	
**Cardiovascular signaling**	3	1	Itga7	Racgap1,Rras2
Complement activation, classical pathway	2	2	C1s,C3	
**Focal adhesion**	7	5	Itga7,Itgb2,Met,Itgb5,Parvb	Itgb6,Akt3
**MAPK signaling pathway**	8	4	Fgfr1,Fgfr2,Rasgrp3,Flnc	Akt3,Nr4a1,Rras2,Fgf11
**B Cell receptor signaling pathway**	6	6	Blnk,Itk,Rps6ka1,Fcgr2b,Ptprc,Rasgrp3	
**Eicosanoid synthesis**	2	2	Ptgds,Ptgs1	
**Signal transduction of S1P receptor**	2	0		Akt3,Racgap1
**OMG vs. YM**	**Total**	**Up**	**Up**	**Down**
Prostaglandin synthesis and regulation	7	6	Ednra,Anxa3,Anxa5,Ptgds,Anxa1,Anxa4	Hsd11b1
Spinal cord injury	11	8	Tnfsf13,Fcgr2a,Tlr4,Il1a,Anxa1,Gfap,Sox9,Vim	Nr4a1,Egr1,Gap43
**Glucuronidation**	5	5	Ugt1a5,Ugt1a6,Ugt1a3,Ugt1a1,Ugt1a2	
Complement activation, classical pathway	4	3	C1s,C1qb,C3	Cd55
Complement and coagulation cascades	7	7	F3,C1qb,C3,Cfh,C4a,A2m,C1s	
Cholesterol metabolism	4	1	Srebf1	Hmgcs1,Lss,Lpl
Serotonin and anxiety	3	1	Plek	Htr2a,Arc
**Estrogen metabolism**	3	3	Ugt1a3,Ugt1a1,Ugt1a2	
**Cholesterol biosynthesis**	3	0		Hmgcs1,Lss,Cyp51
**A Rhodopsin GPCRs, class -like**	12	4	Cmklr1,Gpr34,Ednra,P2ry12	Htr1f,Cnr1,Htr1b,Gpr22,Hcrtr2,P2ry1,Gpr63,Htr2a
Statin pathway	3	2	Lcat,Abca1	Lpl
Hypertrophy model	3	1	Il1a	Nr4a3,Dusp14l1
**Endochondral ossification**	5	4	Fgfr1,Bmp6,Sox9,Stat1	Hmgcs1
**Cytokines and inflammatory response (BioCarta)**	3	3	Il1a,Csf1,Cd4	
Oxidative stress	3	2	Ugt1a6,Mgst1	Junb
**TGF beta signaling pathway**	4	3	Tgfbr1,Smad9,Stat1	Itgb6
**Irinotecan pathway**	2	2	Ugt1a6,Ugt1a1	
**Monoamine GPCRs**	3	0		Htr2a,Htr1f,Htr1b
**Metapathway biotransformation**	7	6	Ugt1a1,Ugt1a2,Ugt1a5,Ugt1a3,Ugt1a6,Mgst1	Cyp51
**p38 MAPK signaling pathway**	3	3	Hspb1,Tgfbr1,Stat1	
**GPCRs, Class C metabotropic glutamate, pheromone**	2	0		Casr,Gabbr2
**OMB vs. OMG**	**Total**	**Up**	**Down**	
Signal transduction of S1P receptor	1	0	Akt3	

*Criterion was ≤-1.5 or ≥1.5-fold change and p ≤ 0.05. Group specific pathway are given in bold. Pathways are arranged descending to significance (from top to bottom) for each group*.

## Discussion

Aging is not necessarily accompanied with cognitive decline. In a cohort of 125 aged male rats, fed with a low energy diet, we found 43.2% males not to be impaired in the holeboard test (RMI ≥0.8). The comparison of the whole cohort with young males show an aging effect in line with other studies (van der Staay et al., 1990). These authors found a age related decline in spatial reference memory in the holeboard with the most profound effect in 19 and 25 month old rats, the age class we used in the present study. However, in spite of the small samples (8–10 rats) also the aged rats in the van der Staay study show an linear improvement in spatial reference memory over blocks of trials, suggesting intact learning capacity in at least some of the rats (unfortunately no standard deviation is given).

Cognitively unimpaired and impaired aged male rats differed in the expression of genes when compared with YM and between the two groups. These genes were related to different pathways or were differently regulated in similar pathways. Genes related to the prostaglandin synthesis and regulation pathway, were regulated with the highest significance in both aged groups compared to YM. Prostaglandine A1 has been shown to protect striatal cells against excitotoxic injuries (Qin et al., 2001). In both aged groups related gene transcripts were mostly up-regulated, however hydroxysteroid 11-beta-dehydrogenase 1-like protein (Hsd11b1) transcript is downregulated in OMG and not regulated in OMB. This gene is involved in the cellular response to estradiol stimulus (Tagawa et al., 2009), glucocorticoid biosynthesis and catabolic processes (Nobel et al., 2002) by 17β-estradiol induced inhibition of the 11 beta-hydroxysteroid hydrogenase, which catalyzes the production of corticosterone from 11-dehydrocorticosterone in rats. The synthesis of Prostaglandines and lipid metabolism are highly interconnected, the latter also with the statin pathway, adipogenesis and eicosanoid synthesis. Lipid synthesis and regulatory pathways are changed in both groups as compared to young rats.

Lipid composition changes in the hippocampus, a brain structure being strongly involved in spatial memory processes, can also lead to specific differences in spatial learning. In a previous study we found nutrition induced lower sphingomyelin amounts in the hippocampal dentate gyrus in rats with impairments of spatial cognitive flexibility (Maliković et al., 2018). The striatum and the hippocampus play different roles in spatial learning and memory (Torromino et al., 2019) and are highly interconnected. The hippocampus projects to the ventral striatum with the nucleus accumbens that has been identified to be critically involved in spatial long-term memory processes (Ferretti et al., 2010; Torromino et al., 2019) with different functions of the core and shell substructures (Meredith and Totterdell, 1999; Ito et al., 2008; Ito and Hayen, 2011). The dorsal striatum in turn contributes to different aspects of spatial memory like egocentric vs. allocentric spatial learning strategies (De Leonibus et al., 2005). These differences cannot be addressed in the present study. However, the extraction of genes and pathways, discriminating between the aged groups, out of thousands of genes, allows the identification of candidates for further, more specific studies.

Previously we showed that also hypothalamic lipid profiles differed between the aged male groups in our paradigm (Wackerlig et al., 2020). The hypothalamus is involved in the regulation of thyroid and steroid hormone release and homeostasis. Interestingly, OMG and OMB mainly differed in the expression of the S1P receptor and in regulatory and functional pathways of steroid hormones. That is glucuronidation, estrogen metabolism and cholesterol biosynthesis. Glucuronidation means the transformation of small lipophylic molecules, like thyroid and steroid hormones, into more water-soluble metabolites. In a previous study we were able to show that blood levels of thyroid hormones, namely triiodothyronine (T3) and thyroid-stimulating hormone (TSH) discriminate aged cognitively impaired from unimpaired male rats (Maliković et al., 2019). It has been shown that treatment with the thyroid hormone triiodothyronine (T3) upregulates the expression of different isoforms of the UDP –glucuronosyltransferase coded by the gene UGT1a6, but decreased Ugt1a1and Ugt1a5, the first was up-regulated by dexamethasone in primary cultures of rats hepatocytes (Li et al., 1999). The up-regulation of all these isoforms here suggests effects of different hormones. Further some isoforms up-regulated here are involved in estrogen metabolism. The synthesis of all steroid hormones is based on cholesterol mainly in the adrenal glands and gonads. Thus, here the downregulation of cholesterol biosynthesis related genes may be a mechanism to reduce tissue cholesterol amount. Estrogen in male rats can locally be synthesized from testosterone by the enzyme aromatase. It has been shown that application of testosterone but not the non-aromatizable dihydrotestosterone improves spatial working memory in aged male rats (Bimonte-Nelson et al., 2003). Hippocampal local activation of estrogen receptors enhances spatial reference memory, in male rats (Meyer and Korz, 2013a,b; Sase et al., 2014). Given the close cooperation of the hippocampus and the striatum this may also have beneficial effects of spatial memory in the striatum. However, while high external estrogen exposure improves memory in hippocampus-dependent tasks, it can impair it in striatum-dependent tasks (Korol and Pisani, 2015). The effects of estrogens upon learning and memory are complex, can be bidirectional (Korol and Pisani, 2015), suggesting the coordinated involvement of different pathways. Post-training corticosterone administration into the dorsomedial striatum has been shown to enhance spatial place memory consolidation in male rats (Lozano et al., 2013).

The TGF Beta signaling pathway specifically changed in OMG vs. YM is involved in multiple cellular signaling processes including cellular homeostasis. TGF-β1restores hippocampal synaptic plasticity and memory in a mouse model of Alzheimer's disease (Hu et al., 2019). The differences in genes related to the MAPK –pathway in OMB and p38 MAPK –pathway in OMG point to different signaling pathways within the MAPK system with cytokine related activation in OMG.

The downregulation of serotonin receptor coding transcripts in both aged groups are probably related to aging, since other studies report similar effects with aging in rats (Wang et al., 1995; Paulose and Balakrishnan, 2008). Functional alterations of the serotonergic system by pharmacological receptor blockade in the striatum of aged male rats has been found to induce spatial learning and memory impairments (Stemmelin et al., 2000; Rutz et al., 2009). Reduced 5-HT2A receptor binding in the striatum of human Alzheimer's disease patients correlated negatively with anxiety and depression scores (Hasselbalch et al., 2008), pointing to functional consequences of striatal serotonin signaling not only for cognition but also mood. Similarily binding of GABAB receptors decrease with age (Milbrandt et al., 1994; Turgeon and Albin, 1994), whereas receptor density remains the same (Turgeon and Albin, 1994). Why this and the calcium sensing receptor coded by Casr are down-regulated only in OMG is unclear, the revelation of functional consequences requires further studies. In OMG these negative effects upon cognition may be compensated by mechanisms mentioned above, whereas in OMB no compensatory processes rescue cognitive impairments.

Such a response is obviously the regulation of the signal transduction of the sphingosine-1-phospate-receptor. This clearly discriminates OMB from OMG as well as from YM. It is a G protein-coupled receptor with the ligand sphingosine-1-phosphate. Striatal S1P is expressed in the striatopallidal neurons in the striatum of mice and essential for instrumental conditiong (Lobo et al., 2007). S1P is involved in a variety of cellular signaling processes including the increase of intracellular calcium from thapsigargin-sensitive stores (Pébay et al., 2001). Pharmacological activation of the S1P receptor by fingolimod increased the levels of brain derived neurotrophic factor (BDNF) and increased the size of the striatum in treated Mecp2 knockout mice as a model of Rett syndrome. BDNF is essentially involved in learning and memory processes and synaptic plasticity (Black, 1999; Yamada et al., 2002; Bramham and Messaoudi, 2005).

Thus, this pathway, among steroid hormone signaling, may significantly contribute to the maintenance of cognitive processes in OMG.

## Data Availability Statement

The datasets presented in this study can be found in online repositories. The names of the repository/repositories and accession number(s) can be found below:

https://gigamove.rz.rwth-aachen.de/d/id/ip2rGzhL4YCFTh, KFB.

## Ethics Statement

The animal study was reviewed and approved by Federal Ministry of Education, Science and Culture, Austria.

## Author Contributions

All authors listed have made a substantial, direct and intellectual contribution to the work, and approved it for publication.

## Conflict of Interest

The authors declare that the research was conducted in the absence of any commercial or financial relationships that could be construed as a potential conflict of interest.
